# Bistatic Radar Configuration for Soil Moisture Retrieval: Analysis of the Spatial Coverage

**DOI:** 10.3390/s90907250

**Published:** 2009-09-10

**Authors:** Nazzareno Pierdicca, Ludovico De Titta, Luca Pulvirenti, Giuliano della Pietra

**Affiliations:** 1 Department of Electronic Engineering, Sapienza University of Rome, via Eudossiana 18, 00184 Rome, Italy; E-Mail: pierdicca@die.uniroma1.it (N.P.); 2 Space Engineering S.p.A., 91, Via dei Berio, Rome 00155, Italy

**Keywords:** bistatic radar, sensors configuration, soil moisture, mission analysis

## Abstract

Some outcomes of a feasibility analysis of a spaceborne bistatic radar mission for soil moisture retrieval are presented in this paper. The study starts from the orbital design of the configuration suitable for soil moisture estimation identified in a previous study. This configuration is refined according to the results of an analysis of the spatial resolution. The paper focuses on the assessment of the spatial coverage *i.e.*, on the verification that an adequate overlap between the footprints of the antennas is ensured and on the duty cycle, that is the fraction of orbital period during which the bistatic data are acquired. A non-cooperating system is considered, in which the transmitter is the C-band Advanced Synthetic Aperture Radar aboard Envisat. The best performances in terms of duty cycle are achieved if the transmitter operates in Wide Swath Mode. The higher resolution Image Swath Modes that comply with the selected configuration have a duty cycle that is never less than 12% and can exceed 21%. When Envisat operates in Wide Swath Mode, the bistatic system covers a wide latitude range across the equator, while in some of the Image Swath Modes, the bistatic measurements, collected from the same orbit, cover mid-latitude areas. In the latter case, it might be possible to achieve full coverage in an Envisat orbit repeat cycle, while, for a very large latitude range such as that covered in Wide Swath Mode, bistatic acquisitions could be obtained over about 65% of the area.

## 1. Introduction

A spaceborne bistatic radar system is defined when antennas for reception and transmission are physically separated and located aboard two spacecraft. The system could be either cooperating (transmitter and receiver designed for the specific bistatic application), or non-cooperating (receiver designed independently of the transmitter) [1]. Non-cooperating systems can use already-existing satellite instruments, such as Synthetic Aperture Radars (SARs), as transmitters (or illuminators) [2]. Bistatic applications were investigated in the fields of target detection [3,4] and planetology [5]. Concerning Earth Observation, the specular reflection from navigation satellites was exploited for scatterometric applications over the oceans [6]. The possibility to envisage bistatic configurations for SAR interferometry was studied in [7,8]. Recently, experiments involving the X-band have been carried out (e.g., [9–11]) to assess the additional information contained in the bistatic reflectivity of targets.

As for land applications, Ceraldi *et al*. [12] demonstrated the potential to detect the signal scattered in the specular direction for retrieving bare soil parameters. However, they outlined that measuring the coherent component of the scattered signal by means of bistatic radars is problematic, because the spatial resolution becomes very poor around the specular direction, and ambiguities between surface points before and after the specular point occur [13]. Zavorotny and Voronovich stated, in a brief report [14], that considering a GPS signal impinging on a rough land surface, soil moisture is related to the ratio between horizontally and vertically polarized scattered waves.

In a previous work [2], we reported on a theoretical investigation aiming at identifying the best bistatic measurement configuration, in terms of incidence angle, observation direction, polarization, and frequency band, for soil moisture content (SMC) retrieval. In addition, we evaluated the improvement of the estimation accuracy with respect to the conventional backscattering measurements. While in [2] we did not tackle the problem of verifying the identified radar configurations from a technical point of view, this work deals with spatial resolution, spatial coverage and duty cycle (*i.e.*, the fraction of the orbit in which suitable bistatic data collection is ensured). It aims at evaluating whether an adequate overlap between the footprints of the transmitting and receiving antennas is ensured for the bistatic observation geometry that guarantees both an improvement of the quality of the SMC retrieval and a quite good spatial resolution.

We point out that investigations on the design of a spaceborne mission implementing a configuration of bistatic radars devoted to specific environmental applications are generally lacking in the literature. Moreover, the literature generally deals with fixed bistatic configurations (*i.e.*, the static design), without considering the orbital dynamics, while the feasibility of maintaining the selected configuration of sensors along the orbit of the spacecraft on which the receiver is installed, ensuring good performances in terms of operational parameters such as spatial coverage, is a crucial aspect.

In the present study, the nominal bistatic mission is considered, so that problems such as the orbit and attitude controls are not tackled, thus implicitly assuming that the passive system is periodically controlled in order to maintain the designed bistatic formation.

In Section 2, the findings regarding the bistatic configurations most suitable for SMC retrieval are summarized and the selection of one configuration, based on the evaluation of the spatial resolution, is described. In Section 3, the orbit design approach is depicted, while, in Section 4, the results of the analysis of the spatial coverage are presented and discussed. Section 5 draws the main conclusions.

## 2. Selection of a Bistatic Configuration

### 2.1. Configurations Suitable for Soil Moisture Retrieval

**Figure 1.**
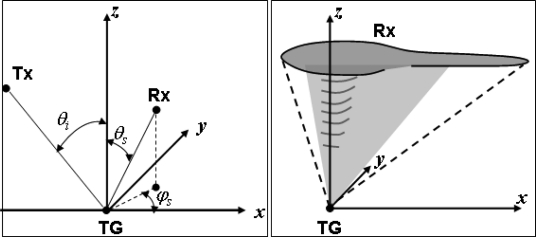
Left panel: geometric elements that identify the transmitter-target-receiver (Tx-TG-Rx) bistatic configuration. Right panel: sketch of observing configurations suitable for SMC retrieval.

Bistatic configurations are defined in terms of frequency, polarization, and transmitter-target-receiver relative geometry. This geometry is shown in the left panel of Figure 1, in which the incident plane is assumed as a reference coordinate plane, and the configuration is identified through the incidence angle (*θ_i_*) with respect to the vertical, the zenith (*θ_s_*) and azimuth (*φ_s_*) scattering angles, and their acceptable ranges to be compatible with the considered application.

In [2], we adopted a well-established electromagnetic surface scattering model, such as the Advanced Integral Equation Model (AIEM) [15], to numerically simulate the bistatic measurements, for the purpose of identifying the configurations suitable for soil moisture estimation. We underline that the AIEM model has a large range of validity, so that the study described in [2] was carried out under different conditions. L-, C- and X-bands were considered, both Gaussian and Exponential autocorrelation functions were adopted, and the parameters characterizing the surface roughness, *i.e.*, the standard deviation of heights *s* and the correlation length *l*, varied within fairly standard intervals, encompassing most of the typical agricultural fields in bare soil conditions. The results we obtained can be therefore considered fairly general.

The findings in [2] indicate that, especially at C-band, the quality of soil moisture retrievals improves by complementing bistatic measurements with monostatic ones provided by already-existing SARs. This could be expected because, for bistatic systems, the detected field is determined by a different behavior of the scattering with respect to the monostatic case, and in the specular configuration also by a coherent scattering mechanism as opposed to a diffuse one, thus providing independent information on the target parameters. The need to take advantage of various sources of information for tackling the complex and usually ill-posed problem of SMC retrieval was indeed pointed out in numerous literature studies (e.g., [16], where polarimetric data were used).

The range of valuable scattering directions, for a C-band radar, singled out in [2] is sketched in the right panel of Figure 1. Observations around nadir, at *θ_s_* below 4°–10°, form a cone (*i.e.*, *φ_s_* can assume any value) whose vertical axis corresponds to the *z*-axis (in a Cartesian reference system), while the vertex is in the origin. Furthermore, for *φ_s_* less than 40°–50°, the zenith scattering angle can indefinitely be increased by including the specular direction and some grazing observations. The illuminator should observe the Earth with a quite small incidence angle, approximately between 15° and 35°. In [2] it was demonstrated that, at C-band, the standard deviation of the SMC retrieval error can be reduced up to a factor 3 with respect to that achievable with monostatic observations, by integrating backscattering and bistatic measurements.

### 2.2. Spatial Resolution Analysis

The ground range and azimuth resolutions could be very poor in some bistatic configurations, thus implying a bad radar image quality. For instance, the ground range resolution is critical in specular configuration [1,12]. Basing on these considerations, we have performed an evaluation of the bistatic spatial resolutions in order to put an additional constraint to the sensor configurations selected in [2]. It is worth noting that no adequate model has been found in the literature for such an exercise, because only few cases addressed the spaceborne receiver configuration. In [1], this matter was analyzed, but only for a two-dimensional (2-D) configuration restricted to the bistatic plane. General considerations and formulas valid for the 3-D case were provided in [13].

Starting from the relationships found in [13], we have derived the formulas for evaluating the ground range (*ρ_gr_*) and azimuth (*ρ_a_*) bistatic resolutions, by doing the following assumptions: *i*) flat and fixed Earth; *ii*) satellites orbiting at the same height (and therefore flying with the same velocity); *iii*) equal coherent integration times. Then, we have normalized the bistatic resolutions to the monostatic ones, obtaining equations depending on the relative geometry of the satellites carrying the sensors and independent from orbital and antenna parameters. In this section, we do not report all the mathematical manipulations, for the sake of conciseness, but only the result. More details can be found in the Appendix. The final equations are the following:

(1) $$\frac{\rho_{\mathrm{gr}}}{{\rho_{\mathrm{gr}}}^{\mathrm{back}}}=\frac{2\;\text{sin}\;\theta_{i}}{\sqrt{{\text{sin}}^{2}\;\theta_{i}+{\text{sin}}^{2}\;\theta_{s}-2\;\text{sin}\;\theta_{i}\;\text{sin}\;\theta_{s}\text{cos}\;\varphi_{s}}}$$

(2a) $$\frac{\rho_{a}}{{\rho_{a}}^{\mathrm{back}}}=\frac{2\;\text{cos}\;\theta_{i}}{\sqrt{F\left(\theta_{i},\theta_{s},\varphi_{s},\delta_{T},\delta_{R}\right)}}$$

where:

(2b) $$\begin{array}{l} F\left(\theta_{i},\theta_{s},\varphi_{s},\delta_{T},\delta_{R}\right)={\text{cos}}^{2}\;\theta_{i}\left({\text{cos}}^{4}\;\theta_{i}\;{\text{cos}}^{2}\;\delta_{T}+{\text{sin}}^{2}\;\delta_{T}\right)+{\text{cos}}^{2}\;\theta_{s}\left[1-{\text{sin}}^{2}\;\theta_{s}\left(1+{\text{cos}}^{2}\;\theta_{s}\right){\text{cos}}^{2}\left(\varphi_{s}-\delta_{R}\right)\right]+\\ +2\;\text{cos}\;\theta_{i}\;\text{cos}\;\theta_{s}\left[{\text{cos}}^{2}\;\theta_{i}\;\text{cos}\;\delta_{T}\;\text{cos}\;\delta_{R}+\text{sin}\;\delta_{T}\;\text{sin}\;\delta_{R}-{\text{sin}}^{2}\;\theta_{s}\left({\text{cos}}^{2}\;\theta_{i}\;\text{cos}\;\delta_{T}\;\text{cos}\;\varphi_{s}+\text{sin}\;\delta_{T}\;\text{sin}\;\varphi_{s}\right)\;\text{cos}\;\left(\varphi_{s}-\delta_{R}\right)\right] \end{array}$$

In Equations (1) and (2a), the *back* superscript indicates the spatial resolutions computed for the conventional monostatic case (*i.e.*, the backscattering measurement) and *δ_T_* and *δ_R_* are the angles complementary to those identified by the unit vector normal to the incidence plane and the velocity vectors (see Appendix).

**Figure 2.**
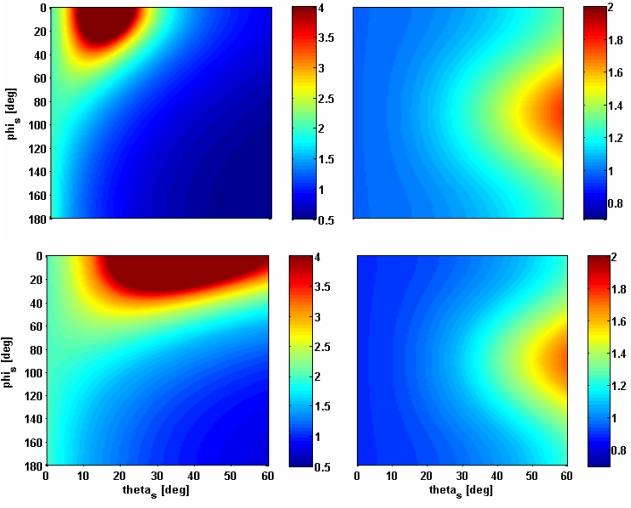
Maps of *ρ_gr_*/*ρ_gr_^back^* (left panels) and *ρ_a_*/*ρ_a_^back^* (right panels), for *θ_i_* = 15° (upper panels) and *θ_i_* = 35° (lower panels) in the (*θ_s_*,*φ_s_*)-plane. Ground range resolution has been upper limited to 4 times the backscattering value for the sake of figure clarity.

Starting from the above formulas, and supposing that the satellites’ velocity vectors are directed normally to the incidence plane (*i.e.*, *δ_T_* = *δ_R_* =90°), maps of *ρ_gr_*/*ρ_gr_^back^* and *ρ_a_*/*ρ_a_^back^* have been generated as function of *θ_s_* (in the range [0°–60°]) and *φ_s_* (in the range [0°–180°]), for a fixed value of *θ_i_*. Figure 2 shows the maps for the minimum and maximum values of *θ_i_* recommended for estimating soil moisture (*i.e.*, 15° and 35°).

By observing Figure 2, it can be noted that the ground range resolution is more critical than the azimuth one. The latter does not exceed 2*ρ_a_^back^*, while *ρ_gr_* can be several times larger than *ρ_gr_^back^*, especially near the specular direction (as expected). As an additional constraint to the bistatic sensors configurations selected in [2], we have chosen *ρ_gr_* < *ρ_gr_^back^*, thus finding that *φ_s_* must be limited in the interval [90°−270°], that is in the backward quadrant (see Figure 1 right panel and the Appendix). We have therefore considered the following ranges for the zenith and azimuth scattering angles: *θ_s_* ∈ [0°−8°] and *φ_s_* ∈ [90°−270°].

## 3. Orbit Design

According to the chosen frequency (*i.e.*, C-band), we have made reference to the ASAR instrument onboard Envisat. The real operative Image Swath Modes (ISMs) and Wide Swath Mode (WSM) have been taken into account for the analysis. ISMs guarantee a fairly high resolution, but have a smaller near and far range capability that implies worse monostatic (and thus bistatic) spatial coverage. WSM presents opposite characteristics. Table 1 reports some parameters of the ASAR illuminator in Image and Wide Swath modes. Note that, among the seven Envisat/ASAR Image Swath Modes, only four were selected, because the other ones observe the Earth with incidence angles larger than 35°, which are not compatible with our requirements for SMC retrieval.

We have firstly designed the orbit of the receiver by choosing its observation geometry at the equator based on the requirements for SMC estimation (*i.e.*, the static design) and then the orbits of both platforms have been propagated to evaluate the antenna footprint superimposition, *i.e.*, the spatial coverage of the bistatic acquisitions. Note that the time when the passive satellite is over the equator has been assumed as the initial one [17].

We have made a number of assumptions. Firstly, that the bistatic system is non-cooperative, so that orbit, attitude (including yaw-steering) and antenna pointing of the active system are not subordinated to the bistatic acquisition requirements. This is of course a worst case assumption, which aims to use existing radar as source of opportunity. Secondly, the transmitting antenna is right-looking. Moreover, the satellite carrying the receiver (hereafter denoted also as the passive satellite) flies in formation with that carrying the illuminator (the active one), on a parallel orbit, thus establishing a “parallel orbit pendulum” configuration, in which the platforms move along orbits with the same inclination, but different ascending nodes. In principle, also the “leader-follower” configuration, in which the spacecraft fly on the same orbit, but with different crossing times of ascending node, would be possible. However, it does not allow any of the bistatic observation geometries previously selected if a non-cooperative bistatic system and a side-looking illuminator are assumed.

Another hypothesis we have made for the static design of the orbits is that the receiver performs bistatic acquisitions only along the ascending pass, since the crossing of the orbits near the poles makes the transmitter to illuminate out of the footprint of the receiver. Note that an eventual left/right looking capability of the receiver would dramatically change the bistatic angles with respect to the required ones, whereas only a cooperative transmitter with left/right looking capability would enable valuable data acquisition in the descending pass.

The orbit of a spacecraft is described by means of the well-known six Keplerian parameters: semi-major-axis, eccentricity, inclination, right ascension of the ascending node (Ω), perigee argument, and mean anomaly (*M*). We have considered Envisat as the active satellite, so that its orbital parameters are fixed. The passive satellite semi-major-axis, eccentricity, and inclination have been chosen coincident with those of the active one, in order to reduce orbit maintenance operations. In addition, the same perigee argument has been considered, to minimize the instantaneous satellite velocity differences and in turn, the along-track relative displacements [17].

**Figure 3.**
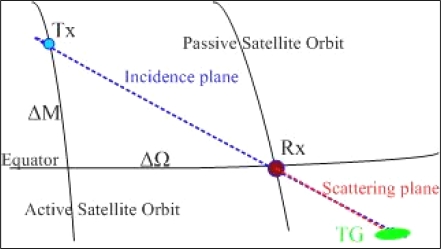
Relative position of the satellites at the initial epoch (γ is the yaw-steering angle of the active satellite).

The remaining design parameters (Ω and *M*) define the difference between the orbits of the two satellites. The differences between the ascending node right ascensions (ΔΩ) and the mean anomalies (Δ*M*), *i.e.*, the relative position of the satellites (shown in Figure 3), have been computed by means of the formulas provided in [17]. Δ*M* has been chosen in order to enable the passive satellite to be located in the incidence plane of the illuminator at the initial time, thus fixing the observation geometry when the receiver is over the equator. Such a condition corresponds to *φ_s_* = 180°, in this case.

Figure 3 shows the position of the active and passive satellites when the latter is over the equator and the line joining the two positions (*i.e.*, the baseline). This direction has to be comprised within the azimuth aperture of both the transmitting and receiving antennas. To calculate ΔΩ and Δ*M*, we have assumed *θ_i_* equal to the maximum among those suggested in [2] (*i.e.*, 35°), in order to increase the range of latitudes for bistatic acquisitions [17]. As for *θ_s_* at the initial epoch, we have firstly considered that the receiver should be in the backward quadrant because of the constraint we have imposed for spatial resolution. Then we have made the hypothesis that, for a soil moisture application, the minimum bistatic swath should be 10 km.

**Figure 4.**
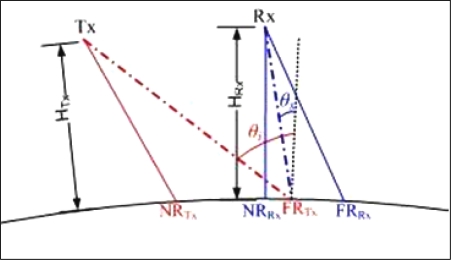
Bistatic observation geometry at the initial time in terms of zenith incidence (*θ_i_*) and scattering (*θ_s_*) angles, receiver (Rx) and transmitter (Tx) near range (NR) and far range (FR) points, and heights (*H*) of the satellites.

By choosing *θ_s_* = 0° at the initial epoch, the time interval during which *θ_s_* is in a useful range (*i.e.*, [0°–8°]) when the orbits are propagated is maximized (note that at the equator the baseline is at its maximum, as will be discussed in Section 4). However, looking at Figure 4, that shows the observation geometry at the initial time, it can be noted that such a choice would imply a very small bistatic swath at the equator (theoretically, only one point), because for *θ_s_* = 0° the receiver is just above the boundary of the area illuminated by Envisat, with the maximum allowable zenith angle (*i.e.*, 35°), and cannot accomplish a forward observation. For the same reason, the ISMs whose far range looking angle is less than 35° (see Table 1) do not perform bistatic acquisitions over the equator, as will be shown in Section 4. The receiving near range point (NR_RX_) has to be located within the area illuminated by the transmitting antenna (see Figure 4) and its distance to the transmitter far range point (FR_TX_) cannot be less than 10 km. Hence, the best choice for the initial *θ_s_* turned out to be 1°, which is (approximately) the minimum zenith scattering angle for which the overlapping between the footprints of the antennas occurs with the selected minimum width of the bistatic swath. Table 2 reports the selected configuration and the orbital parameters at the initial epoch, that is the static design.

The Satellite Tool Kit (available at: http://www.agi.com) has been successively used to propagate the orbits, taking into account only the orbital perturbations due to the *J*_2_ geo-potential harmonic, to maintain a heliosynchronous orbit and assuming negligible the orbital decay (*i.e.*, the effect of the differential aerodynamic drag), as done in [18].

## 4. Discussion of the Outcomes

To produce coverage data, an appropriate software tool, which accounts for spacecraft propagated orbits, yaw-steering maneuvers of both spacecraft, sensors pointing geometry, Earth rotation and estimates the targeted area, has been developed. At each time point of the simulation, whose overall period is 35 days, *i.e.*, the orbit repeat cycle of Envisat, the software evaluates the area of the Earth surface observed simultaneously by the two antennas on the basis of their aperture angles and of the satellites’ positions. The portion of this area which is actually observed according to the observation geometry selected in section 2, *i.e.*, *θ_I_* < 35°, *θ_s_* ∈ [0°−8°] and *φ_s_* ∈ [90°−270°], is the area target. To perform the simulation, we have considered the transmitter able to illuminate according to the real access capabilities of the reference mission (*i.e.*, Envisat, see Table 1). As for the receiving antenna, we have assumed a main lobe azimuth aperture of 6° (see [17]) which is capable to point toward the area illuminated by the transmitter by means of attitude maneuvers or electronic steering. The latter does not ensure the zero Doppler condition of bistatic measurements, but its purpose is the superposition of the antenna footprints.

Table 3 reports the results. It can be noted that WSM presents the best results in terms of duty cycle (∼40%) thanks to the greater access capability. Higher resolution modes achieve duty cycles not less than 12% (actually 11.9%) and that can exceed 21%, which is a still acceptable performance, considering that we are in the hypothesis of a fully non-cooperative system, *i.e.*, the maximum duty cycle is 50% for a fixed looking side of the transmitting antenna. It is worth noting that we have decided to discard the swaths smaller than 10 km in the computation of the fraction of orbital period during which the bistatic data are acquired and this choice implies an overall decrease of the resulting duty cycle.

ISM1 and WSM reach the highest latitudes (almost 72° in both the geographic hemispheres). In addition, the far range capability of the considered transmitter operating mode limits the latitude range of bistatic acquisitions. As stated when discussing Figure 4, since the passive satellite orbit is defined according to the maximum incidence angle identified for the application (*i.e.*, 35°) during static design, the operational modes ISM1, ISM2 and ISM3 do not perform bistatic acquisitions at low latitudes but they focus on mid-latitude belts.

**Figure 5.**
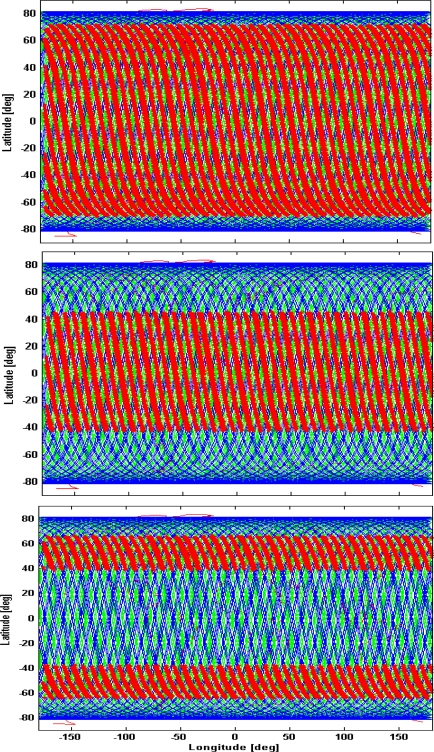
Data acquisition (red dots) for a one week scenario considering a passive system acquiring ASAR signal in WSM (upper panel), ISM4 (central panel) and ISM2 (lower panel). Green/blue lines are the Envisat/passive satellite ground tracks.

Figure 5 shows the Earth maps with the acquired targeted areas (red dots) for WSM (upper panel), ISM4 (central panel) and ISM2 (lower panel). The ground tracks of the active and passive satellites are plotted in green and blue, respectively. Although our simulation considers the entire Envisat orbit repeat cycle (35 days), Figure 5 regards a time-window of one week, for the sake of clarity. The different latitude bands covered in WSM, ISM4 and ISM2 are clearly visible.

To quantify the spatial coverage of the bistatic measurements, we have evaluated the ratio between the sum of the areas of bistatic targets and the area of the Earth surface within the covered latitude range. Such a computation sums up all the areas imaged during a complete Envisat cycle of 35 days, including also zones already observed in previous orbits. This result is therefore an estimate of the fractional coverage that does not ensure that every point within a specific latitude range is actually covered by a bistatic acquisition, but that can indicate whether full coverage could be potentially obtained in such a range. The ratio is minimum for ISM4 and is in the order of 65% for WSM, while for ISM1 and ISM2 our evaluation indicates that full coverage might be achieved within 35 days.

**Figure 6.**
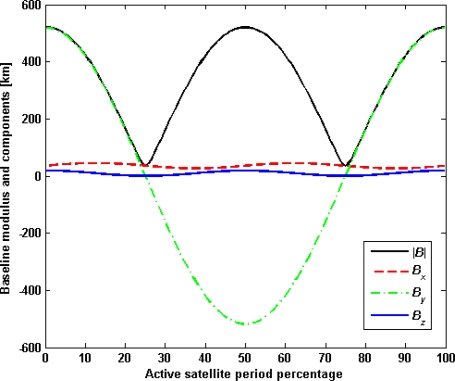
Baseline length (absolute value) and components in the active satellite orbital reference frame versus the orbital period percentage. One orbit is considered for the sake of figure clarity.

Finally, Figure 6 shows the trends of the baseline absolute value |*B*| (distance between the satellites) and of its components (*B_x_*,*B_y_*,*B_z_*) throughout one orbit, that we obtained as a consequence of our design. The baseline is evaluated on the orbital reference frame centered in the centre of mass of the active satellite, with *x*-axis towards its instantaneous velocity vector, *y*-axis normal to the orbital plane and *z*-axis in direction of the Earth center. The distance between the satellites achieves its maximum value at the equator and the minimum one near the poles. *B_y_* represents the main component of *B* and changes its sign at the orbit interceptions near the poles, when the satellites change their relative orientation, while it achieves the maximum value at the equator. Also *B_z_* goes to zero at the orbits interceptions and the minimum baseline at the orbit crossing, *i.e.*, the safety distance between the satellites, has turned out to be equal to 35.5 km, a result similar to that found in [18] in which Envisat was used as the active satellite. Observing Figure 6, it can be noted that the maximum value of the baseline (at the equator), is slightly larger than 500 km.

## 5. Conclusions

Some outcomes of a coverage analysis of a spaceborne bistatic mission for SMC estimation have been presented. The study has started from the identification of the bistatic configurations suitable for SMC retrieval, in terms of frequency and ideal transmitter-target-receiver relative geometry accomplished in a previous study. An evaluation of the spatial resolution of the bistatic system has been firstly carried out. It has led us to restrict our analysis to observations around nadir and in the backward quadrant. Then, the study has assessed the feasibility of the nominal mission in terms of spatial coverage and duty cycle, assuming a non-cooperative system, with Envisat/ASAR as illuminator.

We have shown that the Wide Swath Mode has the best duty cycle (∼40%). Higher resolution image modes yield acceptable results in terms of this parameter (never less than 12%), considering that, in our hypothesis of fully non-cooperative system, the maximum achievable value is 50%. It has been found, that, for high resolution modes, the minimum and maximum latitudes do not necessarily identify one latitude belt, so that their setting may allow focusing on a particular geographic region, such as mid-latitude areas. In a selected latitude band, it might be possible to achieve full coverage within the Envisat orbit repeat cycle (35 days), while for a larger latitude range, almost covering the entire planet, it is unfeasible.

The improvement of soil moisture retrieval that we have demonstrated, in a previous work, to be achievable through a bistatic mission, may be actually obtained by designing a simple and cheap (even in terms of power requirements) receiver that flies in formation with a standard C-band SAR. Combining the active monostatic with the bistatic measurements can strengthen the contribution of single frequency radar observations in many disciplines needing an evaluation of soil moisture, such as hydrology, agriculture and climate change monitoring.

The methodology based on a study of the sensitivity to a geophysical target parameter of bistatic radars, followed by a system performance analysis can be useful for other applications (e.g., vegetation biomass retrieval) and for future missions. For instance, this approach can provide support to a possible employment of a passive receiver flying in formation with the forthcomong European Space Agency (ESA) Sentinel satellites. In particular, the wide swath capability of the Sentinel-1 C-band SAR is expected to improve the coverage performances of the bistatic mission with respect to that computed for Envisat.

## Figures and Tables

**Table 1.** Some parameters of the ASAR illuminator in Image Swath and Wide Swath Modes. Note that both the swath and the ground range resolution refer to the backscattering acquisition; regarding the latter, its value is related to a nominal incidence angle (e.g., 23° for ISM2).

**Mode**	**Swath [km]**	**Near Range [deg]**	**Far Range [deg]**	**Range Resolution [m]**

ISM1	105	15.0	22.9	30
ISM2	105	19.2	26.7	30
ISM3	82	26.0	31.4	30
ISM4	88	31.0	36.3	30
WSM	405	15	37	100

**Table 2.** Selected bistatic configuration (in terms of zenith incidence angle, and zenith and azimuth scattering angles), and orbital parameters at the initial epoch (static design).

zenith incidence angle *θ_i_* [deg]	35
zenith scattering angle *θ_s_* [deg]	1
azimuth scattering angle *φ_s_* [deg]	180
Semi-major-axis [km]	7,159.48
Eccentricity	0.00115
Inclination [deg]	98.5
Perigee argument [deg]	90
Ascending nodes difference (ΔΩ) [deg]	4.20
Mean anomaly difference (Δ*M*) [deg]	0.91

**Table 3.** Coverage statistics and duty cycle, assuming Envisat in different Swath Modes as illuminator. Minimum and maximum acquisition latitudes, and mean width of the bistatic swath (minimum is 10 km) are reported. Note that for ISM1, ISM2 and ISM3, two latitude belts are identified.

**Mode**	**ISM1**	**ISM2**	**ISM3**	**ISM4**	**WSM**

**Duty Cycle [%]**	13.0	12.9	11.9	21.5	40.1
**Lat. Min. 1[deg]**	−71.7	−66.2	−53.2	−43.5	−71.7
**Lat. Max. 1[deg]**	−48.5	−39.6	−24.2	42.9	71.6
**Lat. Min. 2[deg]**	48.9	40.5	25.6		
**Lat. Max. 2[deg]**	71.7	66.2	51.3		
**Swath [km]**	39.9	37.2	29.2	24.1	39.1

**Figure 7.**
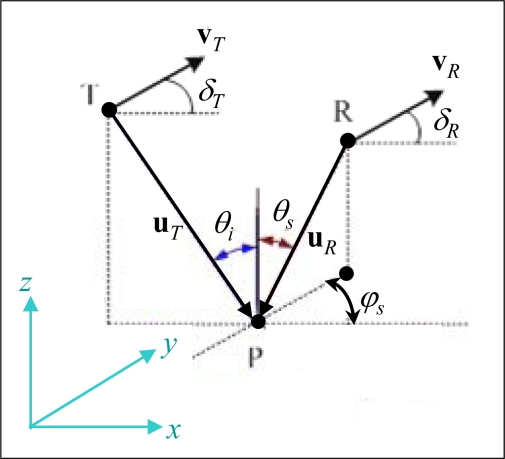
Geometry for evaluating the bistatic spatial resolution. T, R and P represent the positions of transmitter, receiver and target, respectively.

## 6. Appendix Spatial Resolution

In this section, Equations (1) and (2) are derived starting from the relationships found in [13]. The hypotheses of flat Earth and same satellite heights hold true. We make reference to the geometry shown in Figure 7.

By assuming the incidence plane as a reference, let us consider a three-dimensional Cartesian coordinates system in which the (*x*,*z*)-plane is coincident with the incidence one and the (*x*,*y*)-plane is considered to be the (flat) Earth surface. Let us fix: *i*) the transmitter position T (*x_T_*, *y_T_*, *H*) in such a reference frame; *ii*) the incidence angle *θ_i_*; *iii*) the elevation *θ_s_* and azimuth *φ*_s_ scattering angles. Then, we can determine, as function of these parameters, the following ones: *iv*) the ground target position P (*x_T_* + *θ_i_*, 0, 0); *v*) the receiver position R (*x_T_* + *H*tan*θ_i_* + *H*tan*θ_s_*cos*φ_s_*, *y_T_* + *H*tg*θ_s_*sin*φ_s_*, *H*).

Denoting by **x**_0_, **y**_0_ and **z**_0_ the versors of the considered Cartesian system, the vectors directed from the transmitting (**u***_T_*) and receiving (**u***_R_*) antennas to the target are (see Figure 7):

(3) $$u_{T}=H\;\text{tan}\;\theta_{i}x_{0}-Hz_{0}$$

(4) $$u_{R}=-H\;\text{tan}\;\theta_{s}\;\text{cos}\;\varphi_{s}x_{0}-H\;\text{tan}\;\theta_{s}\;\text{sin}\;\varphi_{s}y_{0}-{Hz}_{0}$$

The transmitter-to-target and receiver-to-target ranges are the modules of **u***_T_* and **u***_R_*, respectively:

(5) $$\left|u_{T}\right|=H/\text{cos}\;\theta_{i}$$

(6) $$\left|u_{R}\right|=H/\text{cos}\;\theta_{s}$$

Now we can consider the relationship for the ground range resolution proposed in [13]:

(7) $$\rho_{\mathrm{gr}}=\frac{c}{2B_{W}\left|\Gamma_{\mathrm{xy}}\;u_{\mathrm{eff}}\right|}$$

where *c* denotes the velocity of light, *B_w_* is the pulse bandwidth and Γ*_xy_* is the projector onto the horizontal plane. 2**u***_eff_* is defined as [13]:

(8) $${2u}_{\mathrm{eff}}=-\left(\frac{u_{T}}{\left|u_{T}\right|}+\frac{u_{R}}{\left|u_{R}\right|}\right)$$

Accounting for Equations (3)–(6) it turns out:

(9) $${2u}_{\mathrm{eff}}=\left(\text{sin}\;\theta_{s}\;\text{cos}\;\varphi_{s}-\text{sin}\;\theta_{i}\right)x_{0}+\text{sin}\;\theta_{s}\;\text{sin}\;\varphi_{s}y_{0}+\left(\text{cos}\;\theta_{i}+\text{cos}\;\theta_{s}\right)z_{0}$$

The application of the Γ*_xy_* operator leads to the following relationship:

(10) $${2\Gamma }_{\mathrm{xy}}\;u_{\mathrm{eff}}=\left(\text{sin}\;\theta_{s}\;\text{cos}\;\varphi_{s}-\text{sin}\;\theta_{i}\right)x_{0}+\text{sin}\;\theta_{s}\;\text{sin}\;\varphi_{s}y_{0}$$

By substituting the norm of 2Γ*_xy_* **u***_eff_* in (7), we can derive the final expression of *ρ_gr_*:

(11) $$\rho_{\mathrm{gr}}=\frac{c/B_{W}}{\sqrt{{\text{sin}}^{2}\;\theta_{i}+{\text{sin}}^{2}\;\theta_{s}-2\;\text{sin}\;\theta_{i}\;\text{sin}\;\theta_{s}\;\text{cos}\;\varphi_{s}}}$$

Considering that the ground range resolution of a conventional monostatic radar is *c*/(2*B_w_*sin*θ_i_*), the *ρ_gr_*/*ρ_gr_^back^* ratio is given by (1).

It is worth noting that, if *θ_s_* is equal to 0°, the *ρ_gr_*/*ρ_gr_^back^* ratio does not depend on *φ_s_* and is equal to 2 for every *θ_i_*. Moreover, if we consider *φ_s_* equal to 0° (or around it), when *θ_s_* increases starting from 0°, the *ρ_gr_*/*ρ_gr_^back^* ratio grows too much and is always larger than 2. On the other hand, if we assume *φ_s_* equal to 180° (or around it), when *θ_s_* increases starting from 0°, the *ρ_gr_*/*ρ_gr_^back^* ratio decreases and is always smaller than 2. These considerations can be useful to choose the sensors observation geometry in order to achieve a satisfactory ground range resolution.

To derive the azimuth resolution, we have to consider the transmitting (**v***_T_*) and receiving (**v***_R_*) satellite velocity vectors:

(12) $$v_{T}=v_{T}\;\left(\text{cos}\;\delta_{T}\;x_{0}+\text{sin}\;\delta_{T}\;y_{0}\right)$$

(13) $$v_{R}=v_{R}\;\left(\text{cos}\;\delta_{R}\;x_{0}+\text{sin}\;\delta_{R}\;y_{0}\right)$$

where *v_T_* and *v_R_* are the amplitudes of **v***_T_* and **v***_R_*, respectively, and *δ_T_* and *δ_R_* indicate the angles between these vectors and the incidence plane (see Figure 7).

The expression for the azimuth resolution proposed in [13] is:

(14) $$\rho_{a}=\frac{\lambda }{{2\xi }_{\text{int}}\left|\Gamma_{\mathrm{xy}}\;\omega_{\mathrm{eff}}\right|}$$

where *ξ*_int_ is the time interval during which the target is observed, *λ* is the electromagnetic wavelength and the vector **ω***_eff_* is given by [13]:

(15) $${2\omega }_{\mathrm{eff}}=\frac{v_{\mathrm{Tn}}}{\left|u_{T}\right|}+\frac{v_{\mathrm{Rn}}}{\left|u_{R}\right|}$$

In (15), **v***_Tn_* represents the component of **v***_T_* normal to **u***_T_* and **v***_Rn_* denotes the component of **v***_R_* normal to **u***_R_*. **v***_Tn_* can be expressed as:

(16) $$v_{\mathrm{Tn}}=v_{T}-\left(v_{T}\cdot \frac{u_{T}}{\left|u_{T}\right|}\right)\frac{u_{T}}{\left|u_{T}\right|}=v_{T}-v_{T}\;{\text{sin}}^{2}\;\theta_{i}\;\text{cos}\;\delta_{T}\;x_{0}+v_{T}\;\text{cos}\;\theta_{i}\;\text{sin}\;\theta_{i}\;\text{cos}\;\delta_{T}z_{0}$$

By replacing (12) in (16) it turns out:

(17) $$v_{\mathrm{Tn}}=v_{T}\;{\text{cos}}^{2}\;\theta_{i}\;\text{cos}\;\delta_{T}x_{0}+v_{T}\;\text{sin}\;\delta_{T}y_{0}+v_{T}\;\text{cos}\;\theta_{i}\;\text{sin}\;\theta_{i}\;\text{cos}\;\delta_{T}z_{0}$$

The projection of **v***_Tn_* on the (*x*,*y*)-plane is:

(18) $$\Gamma_{\mathrm{xy}}\;v_{\mathrm{Tn}}=v_{T}\;{\text{cos}}^{2}\;\theta_{i}\;\text{cos}\;\delta_{T}x_{0}+v_{T}\;\text{sin}\;\delta_{T}y_{0}$$

The calculation of **v***_Rn_* is similar to that accomplished to derive **v***_Tn_*:

(19) $$\begin{array}{l} v_{\mathrm{Rn}}=v_{R}-\left(v_{R}\cdot \frac{u_{R}}{\left|u_{R}\right|}\right)\frac{u_{R}}{\left|u_{R}\right|}=v_{R}-v_{R}\;{\text{sin}}^{2}\;\theta_{s}\;\text{cos}\;\varphi_{s}\;\text{cos}\;\left(\varphi_{s}-\delta_{R}\right)x_{0}\\ -v_{R}\;{\text{sin}}^{2}\;\theta_{s}\;\text{sin}\;\varphi_{s}\;\text{cos}\;\left(\varphi_{s}-\delta_{R}\right)y_{0}-v_{R}\;\text{sin}\;\theta_{s}\;\text{cos}\;\theta_{s}\;\text{cos}\;\left(\varphi_{s}-\delta_{R}\right)z_{0} \end{array}$$

By replacing (13) in (19) and by applying the operator that project onto the horizontal plane, it turns out:

(20) $$\Gamma_{\mathrm{xy}}\;v_{\mathrm{Rn}}=v_{R}\left[\text{cos}\;\delta_{R}-{\text{sin}}^{2}\;\theta_{s}\;\text{cos}\;\varphi_{s}\;\text{cos}\;\left(\varphi_{s}-\delta_{R}\right)\right]x_{0}+v_{R}\left[\text{sin}\;\delta_{R}-{\text{sin}}^{2}\;\theta_{s}\;\text{sin}\;\varphi_{s}\;\text{cos}\;\left(\varphi_{s}-\delta_{R}\right)\right]y_{0}$$

By taking into account the relationships (5), (6), (15), (18) and (20), we can determine the projection of the vector 2ω*_eff_*, on the horizontal plane:

(21) $$\begin{array}{l} {2\Gamma }_{\mathrm{xy}}\;\omega_{\mathrm{eff}}=\frac{1}{H}\left\{v_{T}\;{\text{cos}}^{3}\;\theta_{i}\;\text{cos}\;\delta_{T}+v_{R}\;\text{cos}\;\theta_{s}\left[\text{cos}\;\delta_{R}-{\text{sin}}^{2}\;\theta_{s}\;\text{cos}\;\varphi_{s}\;\text{cos}\;\left(\varphi_{s}-\delta_{R}\right)\right]\right\}x_{0}+\\ +\frac{1}{H}\left\{\left[v_{T}\;\text{cos}\;\theta_{i}\;\text{sin}\;\delta_{T}+v_{R}\;\text{cos}\;\theta_{s}\left[\text{sin}\;\delta_{R}-{\text{sin}}^{2}\;\theta_{s}\;\text{sin}\;\varphi_{s}\;\text{cos}\;\left(\varphi_{s}-\delta_{R}\right)\right]\right]\right\}y_{0} \end{array}$$

Having assumed that the two spacecraft fly at the same height, we can make the simplifying hypothesis that the velocity vectors have the same amplitude (*i.e.*, *v_T_* = *v_R_*), thus obtaining:

(22a) $$2\left|\Gamma_{\mathrm{xy}}\;\omega_{\mathrm{eff}}\right|=v_{T}\sqrt{F\left(\theta_{i},\theta_{s},\varphi_{s},\delta_{T},\delta_{R}\right)}$$

where:

(22b) $$\begin{array}{l} F\left(\theta_{i},\theta_{s},\varphi_{s},\delta_{T},\delta_{R}\right)={\text{cos}}^{2}\;\theta_{i}\left({\text{cos}}^{4}\;\theta_{i}\;{\text{cos}}^{2}\;\delta_{T}+{\text{sin}}^{2}\;\delta_{T}\right)+{\text{cos}}^{2}\;\theta_{s}\left[1-{\text{sin}}^{2}\;\theta_{s}\left(1+{\text{cos}}^{2}\;\theta_{s}\right)\;{\text{cos}}^{2}\;\left(\varphi_{s}-\delta_{R}\right)\right]+\\ +2\;\text{cos}\;\theta_{i}\;\text{cos}\;\theta_{s}\left[{\text{cos}}^{2}\;\theta_{i}\;\text{cos}\;\delta_{T}\;\text{cos}\;\delta_{R}+\text{sin}\;\delta_{T}\;\text{sin}\;\delta_{R}-{\text{sin}}^{2}\;\theta_{s}\left({\text{cos}}^{2}\;\theta_{i}\;\text{cos}\;\delta_{T}\;\text{cos}\;\varphi_{s}+\text{sin}\;\delta_{T}\;\text{sin}\;\varphi_{s}\right)\text{cos}\left(\varphi_{s}-\delta_{R}\right)\right] \end{array}$$

The final expression for the azimuth resolution of a bistatic system is derived by replacing (22) in (14):

(23) $$\rho_{a}=\frac{H\;\lambda /\xi_{\text{int}}}{v_{T}\sqrt{F\left(\theta_{i},\theta_{s},\varphi_{s},\delta_{T},\delta_{R}\right)}}$$

The azimuth resolution of a conventional monostatic SAR is given by:

(24) $$\rho_{a}^{\mathrm{back}}=\frac{D}{2}\approx \frac{H\;\lambda /\xi_{\text{int}}}{{2v}_{T}\;\text{cos}\;\theta_{i}}$$

where *D* is the dimension of the antenna. Therefore, the *ρ_a_*/*ρ_a_^back^* ratio turns out to be given by (2a).
